# Incorporating Multimodal Directional Interpersonal Synchrony into Empathetic Response Generation

**DOI:** 10.3390/s25020434

**Published:** 2025-01-13

**Authors:** Jingyu Quan, Yoshihiro Miyake, Takayuki Nozawa

**Affiliations:** 1 Department of Computer Science, Institute of Science Tokyo, Yokohama 226-8502, Japan; quan.j.aa@m.titech.ac.jp (J.Q.); miyake@c.titech.ac.jp (Y.M.); 2 Faculty of Engineering, University of Toyama, Toyama 930-8555, Japan

**Keywords:** affective computing, multimodal learning, empathetic response generation

## Abstract

This study investigates how interpersonal (speaker–partner) synchrony contributes to empathetic response generation in communication scenarios. To perform this investigation, we propose a model that incorporates multimodal directional (positive and negative) interpersonal synchrony, operationalized using the cosine similarity measure, into empathetic response generation. We evaluate how incorporating specific synchrony affects the generated responses at the language and empathy levels. Based on comparison experiments, models with multimodal synchrony generate responses that are closer to ground truth responses and more diverse than models without synchrony. This demonstrates that these features are successfully integrated into the models. Additionally, we find that positive synchrony is linked to enhanced emotional reactions, reduced exploration, and improved interpretation. Negative synchrony is associated with reduced exploration and increased interpretation. These findings shed light on the connections between multimodal directional interpersonal synchrony and empathy’s emotional and cognitive aspects in artificial intelligence applications.

## 1. Introduction

In human conversation, nonverbal information is crucial to judge when and how to generate empathetic responses [1]. As an example, when our partner lowers their gaze; furrows their brows; slightly drops the corners of their mouth; lets their arms hang weakly at their sides; and speaks in a low, slow tone filled with deep helplessness and disappointment, our response should ideally convey more understanding and empathy rather than indifference. Thanks to artificial intelligence, we can identify the speaker’s emotions based on the speaker’s text words [2], facial expressions [3], gestures [4], and tones [5,6], generating an appropriate empathetic response [7,8,9,10]. Such empathetic response generation technologies can be applied to a wide variety of industries, including healthcare chatbots [11], shopping recommendation systems [12], and government analyses of human behavior [13].

However, empathy is complex. In addition to individual information, correlational information between the speaker and responder is also critical [14,15]. Several previous psychological studies have demonstrated that interpersonal synchrony contributes to empathy [16,17]. In particular, empathy is associated with positive synchrony and negative synchrony [16,18,19,20]. We call interpersonal synchrony “directional interpersonal synchrony”, with the distinction between positive and negative directions, operationalizing it using the cosine similarity measure. To the best of our knowledge, directional interpersonal synchrony has been less explored in empathetic response generation. This limited exploration has hindered us from focusing on critical features, optimizing models for improved performance, and deepening our understanding of empathetic response patterns in communication scenarios.

Hence, in our study, we investigate how multimodal directional synchrony contributes to empathetic response generation. We developed a framework based on the Commonsense-aware Empathetic Chatting Machine (CEM) [21]. While the original CEM is a language-based unimodal model, our multimodal model integrates video, audio, and cross-modality directional synchrony to capture rich, interpersonal correlational information in communication scenes. We first evaluated the effectiveness of the proposed model by comparing it to a model without synchrony and to models incorporating different types of synchronies. The results indicate that incorporating directional synchrony significantly improves the appropriateness of generated responses. These findings confirm that directional synchrony was successfully integrated into the model.

Then, our experiments evaluate how ablating different types of directional synchrony influences the generated responses in terms of empathetic communication mechanisms. We found that positive synchrony is associated with generally improved emotion reactions, decreased exploration, and improved interpretation F1-scores. Negative synchrony is also associated with decreased exploration and increased interpretation F1-scores.

Our contributions are summarized as follows:

- We propose a modified CEM that incorporates multimodal directional synchrony.
- The proposed model shows that incorporating multimodal directional synchrony could improve empathetic response generation.
- Our exploratory analysis provides insight into the association between multimodal directional synchrony and the different components of the empathetic communication mechanisms.

## 2. Related Work

### 2.1. Empathetic Response Generation

Empathetic response generation aims to generate an empathetic response. Approaches for this aim are categorized into unimodal and multimodal approaches [6,9,22]. With deep learning technologies, unimodal approaches generate empathetic responses utilizing unimodal textual information [23,24,25,26]. These technologies first tokenize the input text, then feed the token into the encoders to extract features. Given the extracted features, there are two tasks that will be performed. The first task involves emotion classification, while the second involves generating responses. Classifiers were used to obtain emotion labels for the emotion classification task. Decoders were used to generate responses for the response generation task. Empathetic response generation relies on rater’s ratings as ground truth labels. Specifically, gradient descent methods were used to train the decoder to approximate the rater’s ratings for emotion categories.

In contrast to unimodal approaches’ success, multimodal information was often overlooked. The use of visual [27] or audio [28] modalities can also be effective for generating empathy responses in addition to text. In recent years, multimodal approaches have been investigated as a method of developing a better empathetic response generation model with deep learning [6,29,30,31]. For these methods, different networks have been used to extract audio and visual modality features. After that, the multimodality information will be fused to perform the task of emotion classification and response generation. Fusion methods usually refer to the process of concatenation of multiple feature vectors of different modalities into a single feature vector. During the training process, the rater’s rating served as a ground truth label for training different networks to ensure that response attributions (emotion categories and sentiments) coincide as much as possible with the rating of the rater.

Multimodal approaches have demonstrated remarkable success in generating empathetic responses. These methods are, however, insufficient for designing response generation models for human-based communications. Specifically, previous research overlooked interpersonal synchrony for empathetic response generation. Thus, we examined multimodal interpersonal synchrony in this study to generate empathetic responses.

### 2.2. Directional Interpersonal Synchrony

Interpersonal synchrony can be broadly defined as behaviors associated with timing-matching in human interactions [32]. Wynn and Borrie [33] introduced refined definitions of interpersonal synchrony based on class (proximity or synchrony), level (local or global), and dynamicity (static or dynamic). Based on this framework, the concept of synchrony in our study is classified as “Local” and “Static”, and it encompasses both “Proximity” and “Synchrony”. Below is the reasoning.

- “Proximity” refers to the similarity of feature values, while “Synchrony” refers to the similarity of temporal changes in the features [33]. In our study, synchrony is evaluated using the cosine similarity between the features of two interlocutors. Cosine similarity captures both “Proximity” and “Synchrony”. Specifically, “Proximity” involves the similarity of features representing “non-contextual” elements, which do not include temporal changes (e.g., facial expression in one frame), while “Synchrony” captures the similarity of “contextual” features, which account for temporal changes (e.g., body movement in a series of images within a turn). Since our study processes features that include both “contextual” and “non-contextual” elements, it is difficult to classify our approach strictly as “Proximity” or “Synchrony” based on Wynn and Borrie’s framework.
- The level of synchrony refers to the timescale at which synchrony is measured. “Local” is operationally defined as a synchrony between units that are at or below adjacent turns. “Global” means any time scale greater than adjacent turns. We extract synchrony features between adjacent turns in our work. Thus, we should consider our “Interpersonal Synchrony” to be in the “Local” class.
- The concept of “Static” is defined as synchrony without considering statistical changes over time, whereas the concept of “Dynamic” is defined as changes in synchrony over time. In this work, “Interpersonal Synchrony” is defined as a cosine similarity between features from two interlocutors without considering the change in synchrony over time. The concept should be comparable to that of “Static”.

Although “Local Static” synchrony is the closest concept based on previous literature, it is still insufficient to describe interpersonal synchrony in our study. This is because there are two types of interpersonal synchrony: positive and negative [20,34,35]. Specifically, high negative cosine similarity values are difficult to categorize into the conventional “high proximity”, “low proximity”, “high synchrony”, and “low synchrony” categories, as these values reflect the directional aspect of similarity.

Consider a scene where two people walk together [32]. Positive synchrony occurs when the behaviors of two individuals coincide. For example, one person places the right foot forward while the other puts the right foot forward as well. Negative synchrony occurs when two behaviors occur simultaneously but in opposite ways. As an example, one person places his right foot forward while the other places his right foot backwards.

Studies in psychology have examined positive synchrony—such as facial mimicry, body posture mimicry, and speech synchrony—and found that it is linked to empathy [36,37,38]. As an example, positive synchrony has been demonstrated to be related to positive emotions [39]. Although negative synchrony has been studied in the context of human perception of interaction [40,41,42,43], those studies did not explore how negative synchrony relates to empathy. In addition, the above literature is rooted in psychology, whereas in the field of computer science, it is unclear how negative synchrony contributes to empathetic communication in a manner distinct from or similar to positive synchrony.

Recently, interpersonal synchrony has been investigated in AI. It has been demonstrated that interpersonal synchrony is effective for detecting emotional cues for automatic emotion recognition and empathizing with others [44,45]. Although some related works [46,47,48] emphasize interpersonal synchrony in communication between human agents, the use of this technology to generate empathetic text responses that enhance human–human communication are less explored. While some literature has introduced multimodally informed empathetic dialogue generation [49,50,51], these methods often focus solely on text or a combination of audio and text. Visual and cross-modalities have been overlooked. We believe that visual and cross-modal approaches, particularly those incorporating multimodal synchrony for generating empathetic responses, are still underexplored in computer science. Here, we would like to clarify that when we refer to the “empathy” of the generated responses, we mean evaluating the components of empathy within them. The three components considered in this study, which vary depending on the response, are emotional reactions, explorations, and interpretations. These aspects are introduced in the following sections.

### 2.3. Empathy Communication Mechanisms

Empathy has two broad aspects, emotion and cognition [52,53]. Sharma [54] developed empathy communication mechanisms to demonstrate empathy for the responder’s text response. There are three components to empathy communication mechanisms: emotional reactions, interpretations, and explorations.

Emotional Reactions (empathy’s emotion aspect) represent the expression of emotions such as warmth, compassion, and concern, experienced after communicating with the speaker.

Interpretation (empathy’s cognitive aspect) involves communicating an understanding of feelings and experiences derived from the speaker’s words.

Explorations (empathy’s cognitive aspect) represent the improvement of understanding through exploring emotions and experiences not previously communicated.

The three components can be useful in assessing the empathy of the response generated. Previous research [25,55,56], however, focused primarily on emotions as a measure of empathy, ignoring the cognitive dimension. Thus, in this study, we further evaluated the generated response by applying the three empathy communication mechanisms described above.

## 3. Methodology

### 3.1. Problem Definition

**Figure 1:**
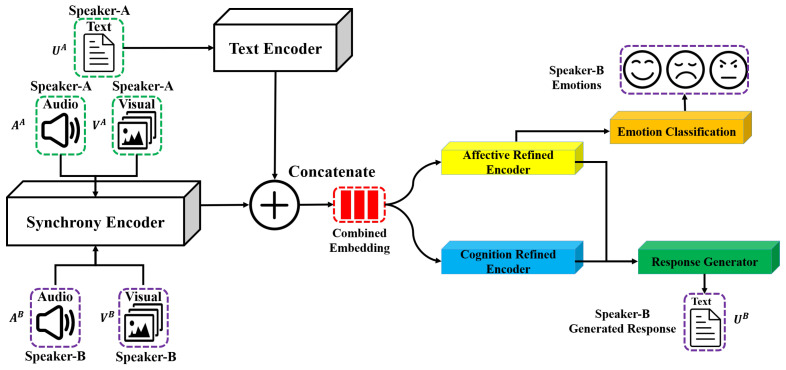
Overview of the incorporation of positive and negative interpersonal synchrony across audio, visual, and cross-modalities to generate empathetic responses.

In this study, we generated empathetic responses using directional interpersonal synchrony in multimodality as features, as shown in Figure 1. The generated responses were evaluated in terms of language and empathy levels. At the language level, we measured the diversity of the generated response and how close it was to the ground truth. At the empathy level, emotion accuracy and empathetic identification (empathy communication mechanisms) were evaluated.

Specifically, during a communication, both Speaker-A and Speaker-B provide visual, audio, and text information. As shown in Figure 1, we generated Speaker-B’s text response with the input of Speaker-A’s speech (converted to text), Speaker-A’s audio and visual information, and Speaker-B’s audio and visual information.

We evaluated the model with the ground truth response along with the emotional label of $Emo_{B}$ (Emotion Classification Label of Response from Speaker-B) and the communication mechanism label, $ER_{B}$ (Emotional Reaction Label), $EX_{B}$ (Exploration Label), and $IP_{B}$ (Interpretation Label).

By creating a model with directional synchrony in multimodality and evaluating its performance, we were able to better understand how they were contributed to emotion and cognitive perception.

### 3.2. Data Preparation

**Figure 2:**
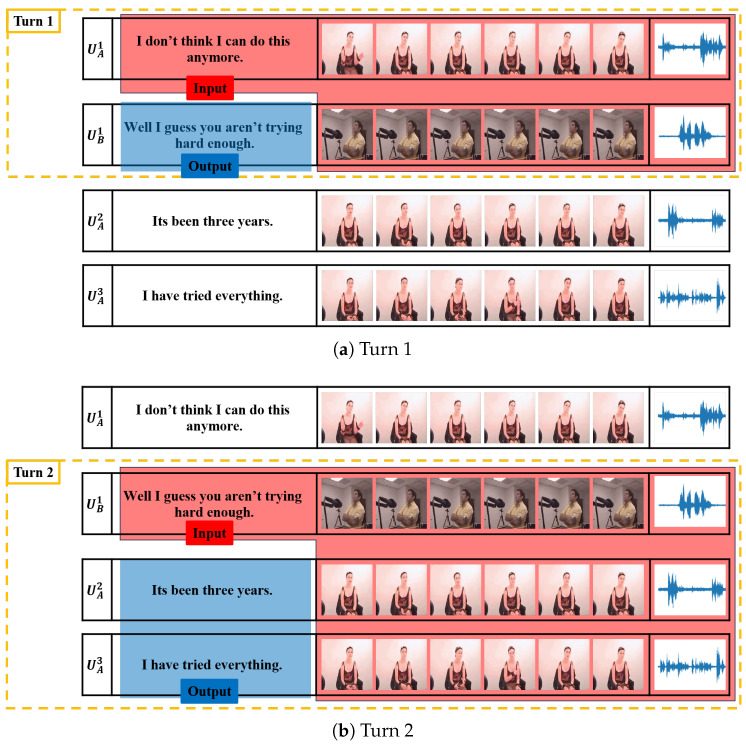
An example of the data using a sliding window method.

Empathetic response generation is conducted by sliding a window of one-turn size over the conversation as shown in Figure 2. Specifically, the window shifts by one turn within the dialogue and the words within the window form a dialogue clip that contains both the input and the response. Using this method, we aim to maximize the use of each utterance in the raw data. We did not take a longer history of utterances into account in order to reduce computational overhead.

As an example, consider the following conversation with utterance indexes of $U_{utterance\;index}^{Speaker}$ from Speaker-A and Speaker-B as $(U_{1}^{A}$,$U_{2}^{A}$,$U_{3}^{B}$,$U_{4}^{B}$,$U_{5}^{B})$. This conversation is divided into turns as $\left(T^{A},T^{B}\right)$. Here, $T^{A}=\left(U_{1}^{A},U_{2}^{A}\right)$ is the context for our task. $T^{B}=\left(U_{3}^{B},U_{4}^{B},U_{5}^{B}\right)$ is the ground truth response. For visual and audio information, we have $V^{A}=\left(V_{1}^{A},V_{2}^{A}\right)$ for speaker-A visual information and $A^{A}=\left(A_{1}^{A},A_{2}^{A}\right)$ for speaker-A audio information. Also for Speaker-B, we have $V^{B}=\left(V_{3}^{B},V_{4}^{B},V_{5}^{B}\right)$ for speaker-B visual information and $A^{B}=\left(A_{3}^{B},A_{4}^{B},A_{5}^{B}\right)$ for speaker-B audio information. As a summary, for the multimodal empathetic response generation task, we have $T^{A}$, $V^{A}$, $V^{B}$, $A^{A}$, and $A^{B}$ as the input data. $T^{B}$ is our target response. Each target response had an emotion label and a communication mechanism label attached. Emotion labels were chosen from the most frequent emotion labels in utterances $T^{B}=\left(U_{3}^{B},U_{4}^{B},U_{5}^{B}\right)$. If there was no single most frequent emotion label, we picked the emotion label of $U_{3}^{B}$, as it is the closest response of Speaker-B to Speaker-A. Empathy communication mechanism labels were annotated using a pretrained classification model [54] for empathetic identification with ground truth responses $T^{B}=\left(U_{3}^{B},U_{4}^{B},U_{5}^{B}\right)$ as input.

In our study, the alignment of the different modalities is definitely taken into consideration. In particular, textural data are represented by an utterance-level annotation, and we are able to obtain timestamps for the audio and visual modalities. As a result of these timestamps, we were able to clip the original audio and visual images and align them according to the timestamps, resulting in the alignment of the different modality data.

### 3.3. Model Creation

#### 3.3.1. Backbone Model Introduction

Our baseline model is based on a popular method called CEM (Commonsense-aware Empathetic Chatting Machine) [21]. CEM was chosen as our backbone because it included both affective and cognitive encoders to deal with the original text. As CEM generated empathetic responses incorporating both cognitive and emotional information, annotating the generated response with Emotional reaction, Exploration, and Interpretation makes sense.

Previous CEM focused solely on text and did not incorporate multimodal information. Further, it did not take into account interpersonal synchrony-related information that influences empathetic responses. Additionally, despite the fact that the original CEM [21] evaluated how cognitive encoders and affective encoders affect emotion accuracy automatically, the evaluation can only reflect empathy’s emotional aspect. Empathy’s cognitive aspect has not been adequately evaluated automatically.

In our study, we extended the original CEM into a multimodal framework. We further incorporated interpersonal synchrony and evaluated the model in terms of both emotion and cognition aspects, as shown in Figure 1.

#### 3.3.2. Individual Feature Encoding

**Figure 3:**
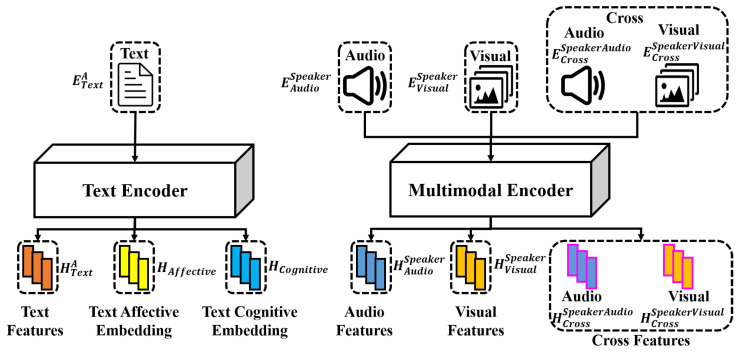
Extraction of individual features for Speaker-A and Speaker-B in audio, visual, and text modalities.

In this study, our goal is to generate the response text for Speaker-B responding to Speaker-A, incorporating directional interpersonal synchrony. Our first step was to collect individual features. The individual features include three modalities: textual modality, visual modality, and audio modality. In particular, we extracted text representations for Speaker-A’s text as well as audio and visual representations for Speaker-A and Speaker-B, as shown in Figure 3.

For the text modality, following the previous work [21], Speaker-A’s utterances in the dialogue history are concatenated and prepended with a special token [CLS] to obtain the text input $T^{A}=\left[CLS\right]⊕U_{1}^{A}⊕U_{2}^{A}⊕\cdots ⊕U_{k-1}^{A}$. Given the sequence $T^{A}$, we sum up the word embedding and positional embeddings as embedding $E_{Text}^{A}$. The sequence embeddings $E_{Text}^{A}$ were then fed into a text encoder to produce the text representation $H_{Text}^{A}$, as shown in Figure 3.

Based on previous work [57] dealing with the IEMOCAP dataset, 3D-CNN and openSMILE were used for extracting visual and acoustic features, respectively. Specifically, our features include visual embeddings from Speaker-A ($E_{Visual}^{A}$), visual embeddings from Speaker-B ($E_{Visual}^{B}$), audio embeddings from Speaker-A ($E_{Audio}^{A}$), and audio embeddings from Speaker-B ($E_{Audio}^{B}$). The cross embeddings consist of both audio and visual embeddings for Speaker-A and Speaker-B, namely, $E_{Cross}^{AAudio}$, $E_{Cross}^{AVisual}$, $E_{Cross}^{BAudio}$, and $E_{Cross}^{BVisual}$. The cross embeddings are calculated separately, as in the next step, both audio–visual and visual–audio interpersonal synchrony features are extracted for Speaker-A and Speaker-B (see Section 3.3.3 below). We note that $E_{Cross}^{AAudio}$ is a copy of $E_{Audio}^{A}$, as well as for the other cross-modal embeddings.

#### 3.3.3. Interpersonal Synchronization Encoding

**Figure 4:**
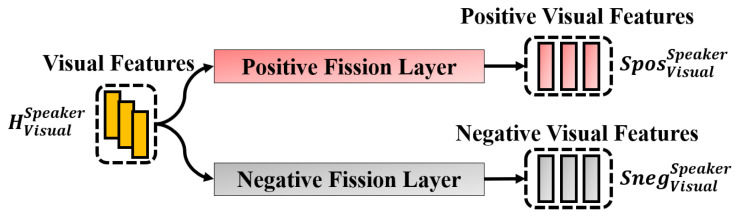
Extraction of positive and negative synchrony-related features for Speaker-A and Speaker-B in visual modality. The same fission procedure is also applied to audio and cross modalities.

**Figure 5:**
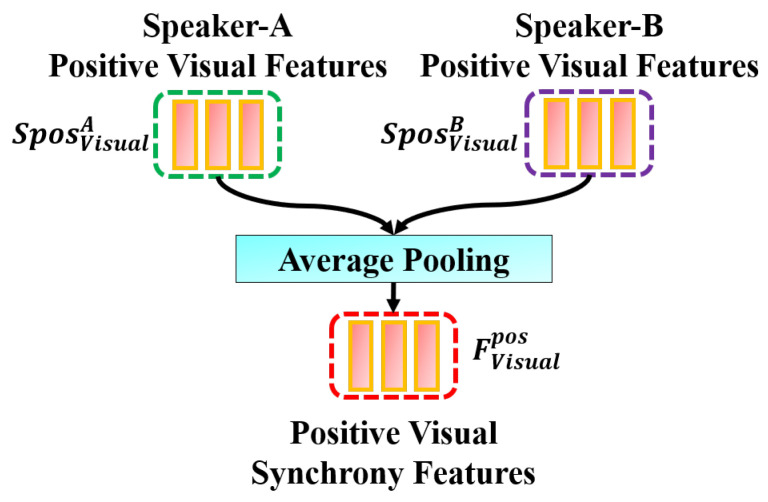
Procedure to obtain visual synchrony features for Speaker-A and Speaker-B. A similar procedure is also applied to obtain the synchrony features for audio and cross-modalities.

Based on the individual features of Speaker-A and Speaker-B for visual and audio modalities, we extracted interpersonal synchrony as shown in Figure 4 and Figure 5. Despite the fact that numerous methods [44,45] are available to extract interpersonal synchrony, such as the calculation of cosine similarity between two features, these methods generally extract the coarse synchrony. This means that the extracted synchrony reflects the entire situation. As an example, both Speaker-A and Speaker-B smile at the same time, which indicates positive synchrony of the head parts. Their body synchrony, however, is negative since they move in opposite directions simultaneously. The cosine similarity between Speaker-A and Speaker-B may provide relatively low positive synchrony. However, the specific positive and negative values are missing. In this study, we extracted positive and negative synchrony separately for each modality. By separating these two directions of synchrony, we can also explore how they affect empathy’s cognitive and emotional aspects differently.

For the visual modality, as shown in Figure 3, we used encoders based on the transformer framework [58] to extract the hidden representation ($H_{Viusal}^{A},H_{Visual}^{B}$) using visual embedding ($E_{Visual}^{A},E_{Visual}^{B}$) as input for Speaker-A and Speaker-B separately.

The challenge is to extract positive and negative synchrony features separately. To accomplish this, we used the fission layer (Figure 4) to extract positive and negative features.

As shown in Figure 4, given $H_{Visual}^{A}$ and $H_{Visual}^{B}$, we fed them separately into a layer to fission the original features to obtain $Spos_{Visual}^{A}$, $Spos_{Visual}^{B}$, $Sneg_{Visual}^{A}$, and $Sneg_{Visual}^{B}$. This fission layer is constructed using Multi-Layer Perceptrons (MLPs), which can extract related information based on the constraints provided. After obtaining $Spos_{Visual}^{A}$ and $Spos_{Visual}^{B}$, we fed them into the average pooling layer to obtain the positive synchrony feature ($F_{Visual}^{pos}$), as shown in Figure 5. Similarly, the negative synchrony feature ($F_{Visual}^{neg}$) is obtained by feeding $Sneg_{Visual}^{A}$ and $Sneg_{Visual}^{B}$ into the average pooling layer.

**Figure 6:**
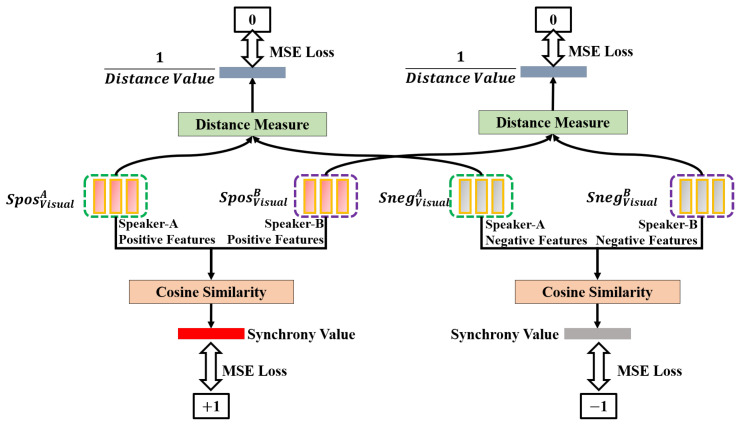
The constraint in the extraction of features related to positive and negative synchrony.

Now, we explain the detail of the constraints that are used in the fission layer. As shown in Figure 6, we calculated the cosine similarity between $Spos_{Visual}^{A}$ and $Spos_{Visual}^{B}$ to obtain $Sync_{V}^{P}$ and calculated the cosine similarity between $Sneg_{Visual}^{A}$ and $Sneg_{Visual}^{B}$ to obtain $Sync_{V}^{N}$. Finally, we constrained $Sync_{V}^{P}$ to be close to 1, while $Sync_{V}^{N}$ was constrained to be close to −1 in Equation (1). In the meantime, we calculated the distance between $Spos_{Visual}^{A}$ and $Sneg_{Visual}^{A}$ as $Dis_{Viusal}^{A}$. We also calculated the distance between $Spos_{Visual}^{B}$ and $Sneg_{Visual}^{B}$ as $Dis_{Viusal}^{B}$. We constrained $Dis_{Viusal}^{A}$ and $Dis_{Viusal}^{B}$ to be as large as possible, as shown in Equation (1). Finally, $Sync_{V}^{P}$, $Sync_{V}^{N}$, $Dis_{Viusal}^{A}$, and $Dis_{Viusal}^{B}$ were combined as a loss ($L_{Visual}^{Sync}$), shown in Equation (1), to control the feature extraction process.

(1) $$\begin{array}{cc} Dis_{Viusal}^{A} & ={\left(Spos_{Viusal}^{A}-Sneg_{Viusal}^{A}\right)}^{2}\\ Dis_{Viusal}^{B} & ={\left(Spos_{Viusal}^{B}-Sneg_{Viusal}^{B}\right)}^{2}\\ L_{Visual}^{Sync} & ={\left(Sync_{V}^{P}-1\right)}^{2}+{\left(Sync_{V}^{N}-\left(-1\right)\right)}^{2}+{\left(\frac{1}{Dis_{Visual}^{A}}\right)}^{2}+{\left(\frac{1}{Dis_{Visual}^{B}}\right)}^{2} \end{array}$$

Here, we note that positive synchrony represents synchrony, when the cosine similarity between Speaker-A and Speaker-B’s features is close to 1, which indicates that the features are very similar (e.g., Speaker-B’s smile reacted to Speaker-A’s smile). Conversely, negative synchrony represents synchrony, when the cosine similarity between Speaker-A and Speaker-B’s features is close to −1, which implies that the features are opposite (e.g., Speaker-B’s smile reacted to Speaker-A’s cry).

We also performed the same operation for audio and cross-modalities, as shown in Figure 3, Figure 4 and Figure 5. For the audio modality, it is the same as with visual modality. For cross-modality, we used encoders based on the transformer framework to extract the hidden representation ($H_{Cross}^{SpeakerVisual},H_{Cross}^{SpeakerAudio}$) using visual embedding ($E_{Cross}^{SpeakerVisual}$) and audio embedding ($E_{Cross}^{SpeakerAudio}$) as input for Speaker-A and Speaker-B separately. We note that $H_{Cross}^{SpeakerVisual}$ is different from $H_{Visual}^{Speaker}$ as they used different encoders and were controlled by different losses.

This design is based on our assumption that important synchrony features within each modality (audio and visual) differ across modalities. For instance, within the visual modality, key correlations might involve both interlocutors’ facial expressions (e.g., both smiling). However, across audio and visual modalities, the important correlation could be between gestures (rather than facial expressions) and the tone of speech, as humans sometimes display fake smiles. This further explains why we require cross-modality instead of relying solely on either visual or audio modalities.

Next, we calculated both audio–visual and visual–audio features for Speaker-A and Speaker-B. Namely, we obtained the following:

- Positive related features:
  $Spos_{Cross}^{AVisual}$, $Spos_{Cross}^{AAudio}$, $Spos_{Cross}^{BVisual}$,$Spos_{Cross}^{BAudio}$
- Negative related features:
  $Sneg_{Cross}^{AVisual}$,$Sneg_{Cross}^{AAudio}$, $Sneg_{Cross}^{BVisual}$,$Sneg_{Cross}^{BAudio}$

Based on the above features, we calculated cross loss ($L_{Cross}^{Sync}$) using Equations (2) and (3).

(2) $$\begin{array}{cc} Sync_{C}^{PM_{A}M_{B}} & =CosineSimilarity(Spos_{Cross}^{M_{A}},Spos_{Cross}^{M_{B}})\\ Sync_{C}^{NM_{A}M_{B}} & =CosineSimilarity(Sneg_{Cross}^{M_{A}},Sneg_{Cross}^{M_{B}})\\ Dis_{C}^{M} & ={\left(Spos_{Cross}^{M}-Sneg_{Cross}^{M}\right)}^{2}\\ \left(M_{A},M_{B}\right) & \in \{(AAudio,BVisual),(AVisual,BAudio)\}\\ M & \in \{AAudio,BVisual,AVisual,BAudio\} \end{array}$$

(3) $$\begin{array}{cc} L_{Cross}^{Sync} & =\sum_{M}\left[{\left(Sync_{C}^{PM_{A}M_{B}}-1\right)}^{2}+{\left(Sync_{C}^{NM_{A}M_{B}}+1\right)}^{2}+{\left(\frac{1}{Dis_{C}^{M}}\right)}^{2}\right] \end{array}$$

As shown in Equation (4), we obtained $F_{Cross}^{pos}$ by feeding positive related features into the average pooling layer. We obtained $F_{Cross}^{neg}$ by feeding negative related features into the average pooling layer.

(4) $$\begin{array}{cc} F_{Cross}^{pos}=AveragePooling( & Spos_{Cross}^{AVisual},Spos_{Cross}^{AAudio},\\ & Spos_{Cross}^{BVisual},Spos_{Cross}^{BAudio})\\ F_{Cross}^{neg}=AveragePooling( & Sneg_{Cross}^{AVisual},Sneg_{Cross}^{AAudio},\\ & Sneg_{Cross}^{BVisual},Sneg_{Cross}^{BAudio}) \end{array}$$

#### 3.3.4. Model Frameworks

By extracting individual and interpersonal similarity features, it is simple to extend the original CEM to incorporate visual, audio, and cross stimuli in the textual modality. This is achieved by combining all the features through a concatenation operation, as shown in Figure 1. After the concatenation operation, we adjust the decoder layer input size to make it adapt to the combined multimodal features. Following is a detailed explanation of how and what modality-related information and directional synchrony features were combined.

For the visual modality, the contextual features $H_{CTX}^{Visual}$ were obtained by combining individual features ($H_{Text}^{A},H_{Visual}^{A},H_{Visual}^{B}$), positive synchrony features ($F_{Visual}^{pos}$), and negative synchrony features ($F_{Visual}^{neg}$). It should be noted that the combining operation here is the concatenation operation, which is used to join a sequence of vectors along an axis.

(5) $$\begin{array}{cc} H_{CTX}^{Visual} & =H_{Text}^{A}⊕H_{Visual}^{A}⊕H_{Visual}^{B}⊕F_{Visual}^{pos}⊕F_{Visual}^{neg} \end{array}$$

For the audio modality, same as the visual modality, the contextual features were obtained by combining individual features ($H_{Text}^{A},H_{Audio}^{A},H_{Audio}^{B}$), positive synchrony features ($F_{Audio}^{pos}$), and negative synchrony features ($F_{Audio}^{neg}$). Same as the visual modality, we used the concatenation operation.

For the cross-modality, the contextual features were obtained by combining individual features ($H_{Text}^{A}$, $H_{Visual}^{A}$, $H_{Visual}^{B}$, $H_{Audio}^{A}$, $H_{Audio}^{B}$, $H_{Cross}^{AVisual}$, $H_{Cross}^{AAudio}$, $H_{Cross}^{BVisual}$, $H_{Cross}^{BAudio}$), positive synchrony features ($F_{Visual}^{pos}$, $F_{Audio}^{pos}$, $F_{Cross}^{pos}$), and negative synchrony features ($F_{Visual}^{neg}$, $F_{Audio}^{neg}$, $F_{Cross}^{neg}$). Same as the visual and audio modalities, we used the concatenation operation.

Following the original CEM [21], we extracted emotion-related knowledge and cognition-related knowledge as $h_{xReact}$ and $h_{r}$ with the knowledge acquisition model shown in Figure 3. To refine the context by adding additional information for emotion and cognition aspects, respectively, we also feed the combination of $h_{xReact}$ and $H_{CTX}^{modality}$ together into the affective refined encoder (shown in Figure 1) to obtain $H_{Affective}$. We feed the combination of $h_{r}$ and $H_{CTX}^{modality}$ into the cognitive refined encoder (shown in Figure 1) to obtain $H_{Cognitive}$. The affective refined encoder and the cognitive refined encoder are the same as original CEM [21] using the transformer framework [58].

**Emotion Classification Task:** As shown in Figure 1, we fed the output from the Affective-Refined Encoder into the Emotion Classification Model to classify the emotion. Specifically, given the $H_{Affective}$, the original CEM used the hidden representations of the specific token [CLS] to classify user’ emotions. However, different from the original CEM focused on the user’s emotion (Speaker-A), in this study we hope to focus more on the generated response and our emotion label is Speaker-B’s emotion label. Namely, all information in $H_{Affective}$ are correlated with the responder’s generated response. Therefore, we performed average pooling to obtain a summarized representation. Finally, the summarized representation was fed into the linear layer and SoftMax layer to perform the emotion classification task.

**Response Generation Task:** As shown in Figure 1, we fed the output from Affective-Refined Encoder and Cognition-Refined Encoder into the Response Generator Model to generate the response (Speaker-B). To generate more appropriate responses, following the original CEM [21], we performed Knowledge Selection to combine $H_{Affective}$ and $H_{Cognitive}$ and fed the combination into a Multi-Layer Perceptron with ReLU activation to obtain the refined contextual representation $F_{CTX}^{modality}$. Finally, we fed the contextual representation into the decoder to generate the response. We noted that the response generator model is the same with original CEM [21].

### 3.4. Evaluation Metrics

The generation model is evaluated at two levels: Language-Level Evaluation and Empathy-Level Evaluation. Specifically, we evaluate the generation model using BLEU scores [59], Rouge-n [60], and Distinct-n [61] at the language level.

The BLEU represents the overlap between the generated response and the ground truth. The higher the BLEU, the closer the generated response is to the ground truth (the highest is 100).

The ROUGE-n also represents overlap between the generated response and the ground truth. The difference between the ROUGE method and the BLEU method is that the BLEU method focuses on precision (mapping the generated response to the ground truth) while the ROUGE method focuses on recall (mapping the ground truth to the generated response). Our study used ROUGE-1 and ROUGE-2. ROUGE-1 evaluated accuracy while ROUGE-2 evaluated fluency.

Distinct-n measures the proportion of unique n-grams in the generated responses and is often used to evaluate generational diversity.

On the empathy level, we evaluated the accuracy of emotion classification [21] for responders and empathy identification accuracy [54] based on communication mechanisms.

Emotional Accuracy (EA) represents whether the responder’s emotion coincides with the ground truth or not.

Furthermore, we evaluated the classification results using accuracy and F1-score with respect to Emotion Reaction (ER), Exploration (EX), and Interpretation (IP). Specifically, we measured whether the generated response was classified the same as the ground truth in terms of with or without emotional reactions, explorations, and interpretations, respectively (see Section 4.1 below for the definition of “with” and “without”).

The accuracy of Emotion Reaction may reflect empathy’s emotional aspect. The Exploration accuracy and Interpretation accuracy could indicate the cognitive aspect of empathy for the generated response.

## 4. Experiment Results and Discussion

### 4.1. Dataset Preparation

We conducted our experiment on an established emotional dialog dataset, IEMOCAP [62]. Other datasets, such as K-EmoCon [63], IFADV [64], and CANDOR [65], were not used due to their limitations for our study. Specifically, the K-EmoCon data contain 10-min debates. However, the number of conversation turns is very limited. In each 10-min debate, only three or four turns may occur. Since our research focuses on response generation, K-EmoCon’s limited turns make it unsuitable for this task. The IFADV corpus is another excellent dataset. However, to our knowledge, its annotations include orthographic transliteration, POS tagging, word alignment, word-to-phoneme alignment, phoneme alignment, conversational function, and gaze direction. Unfortunately, it lacks high-quality utterance-level emotion annotations, which are essential for our study. CANDOR is also a valuable dataset with dyadic conversations. However, to our knowledge, the majority of CANDOR dataset conversations are conducted via video chat, either computer-to-computer or using mobile devices. These settings differ from the face-to-face, offline dyadic conversations that are the focus of this study, making the dataset unsuitable for our purposes.

The IEMOCAP dataset contains approximately 12 h of audio, visual, and text data. It includes dyadic conversations by five dyads (10 speakers), with each dyad having one session of conversation. We used the first four sessions of the dataset for training and validation. The last session was used for testing. This study aimed at finding generalization results. The partitions of the dataset had no shared speaker. Our experiments focused on six categories of emotions including Happiness, Sadness, Neutrality, Anger, Excitement, and Frustration.

We segmented the IEMOCAP dataset conversation data into data units (i.e., turns). The procedures for data segmentation and data labeling are described in Section 3.2 above. After segmentation, we obtained 3750 turns of training and validation data and 1118 turns of test data. As shown in Table 1, for the training and validation data, each turn takes approximately 6.65 s and contains approximately 21.74 words and 1.44 utterances on average. For the test data, each turn takes approximately 6.36 s and contains approximately 22.13 words and 1.39 utterances on average.

As explained in Section 3.2, (pseudo-)ground truth labels for the empathy communication mechanism of each response were assigned using a pretrained classification model [54]. The original pretrained classification model returns three classes—0 (no), 1 (weak), and 2 (strong)—for each of emotional reactions, explorations, and interpretations. However, the distribution of these three classes was remarkably uneven when applied to our data. The train set in pretrained data, for example, contained 53.59% “no” labels, 3.63% “weak” labels, and 41.77% percent “strong” labels. To mitigate the unevenness between classes, we merged 1 (weak) and 2 (strong) into with. Thus, the ground truth responses were annotated with 0 (without) or 1 (with) for each of emotional reactions, explorations, and interpretations.

### 4.2. Implementation Details

Our study utilizes all multimodality information (i.e., visual and audio information from both Speaker A and B, plus text data from Speaker A) to generate Speaker B’s text response (Figure 1), while also determining how various inputs influence the generated response. Specifically, the textual data include Speaker A’s statement in a turn. Audio data consist of recorded interlocutors’ speech, represented as time series data. Visual data consist of interlocutors’ images in a turn. The model output includes two parts. The first part is the textural response. In this case, Speaker B’s textural response is used as the ground truth response. The second part is the emotion annotation of the response such as happiness, sadness, etc.

Following the previous study [21], we implemented all the models using PyTorch (version 1.13.1) and used 300-dimensional pretrained GloVe vectors to initialize the word embeddings, which were shared between the encoders and the decoders. For the audio modality, the raw audio was processed using openSMILE [66] to obtain 100 dimensional vectors for each utterance duration. For the visual modality, the raw images were processed using 3D-CNN [67] to obtain 128 dimensional vectors for each utterance duration.

For the training, Adam was used as the optimizer. The initial learning rate was 0.0001. All the models were trained on a single RTX 3090 GPU (Nvidia Corp., Santa Clara, CA, USA) with a batch size of 16 and early stopping. We used a batch size of 1 and a maximum of 50 decoding steps during testing and inference. The large batch size used during training primarily serves to accelerate the training process and ensure more stable and accurate gradient estimates. On the other hand, during testing, we use a batch size of 1 to facilitate the independent evaluation of each generated response. Specifically, using a batch size of 1 allows us to assess each response sentence individually, which is crucial for evaluation metrics like BLEU. These metrics require evaluating each case separately rather than in batches to provide a more precise assessment of the model’s performance. It is noted that 20% of the training set was used as a validation set when tuning hyperparameters.

### 4.3. Empathetic Response Generation Using Multimodality: Effectiveness Test

Table 2 presents baseline results without incorporating interpersonal synchrony in different modalities. The comparison between text-only MIME and text-only CEM shows that CEM is an effective method for establishing a baseline. When different modality information was added to the generation model, the model outperformed the original text modality model in terms of all metrics. BLEU and ROUGE metrics demonstrate that the generated responses are more close to the ground truth. The Distinction metrics show that multimodality information can be used to generate more diverse responses. Additionally, the EA metric indicates that the multimodal features used in the model improve recognition of the responder’s emotions. This means the generated responses are emotionally more accurate than those that only incorporate text. These results indicate that multimodal information has been successfully incorporated into the model.

### 4.4. Empathetic Response Generation Using Directional Synchronizations: Effectiveness Test

**Figure 7:**
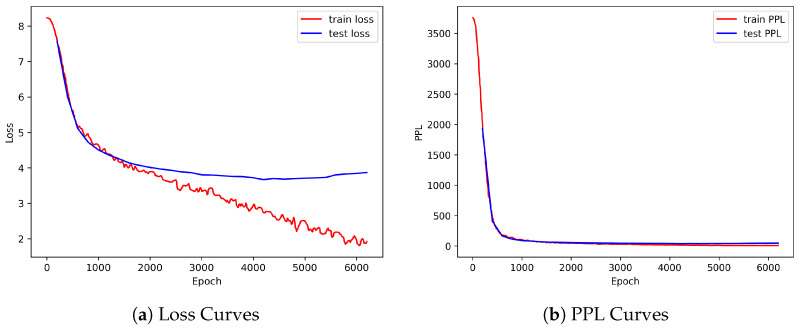
Visualization of the training and test loss and PPL curves for cross-modality with both positive and negative synchrony.

Figure 7 illustrates the training and test loss and perplexity (PPL) curves for cross-modality with both positive and negative synchrony. The learning curves were similar for other models with different modalities and types of synchrony. As shown in Figure 7a, the training loss continues to decrease, while the test loss initially decreases, then stabilizes, and finally begins to rise slightly. The relatively high test loss in comparison with the training loss may raise concerns that the model is not generating proper responses. To address this, Figure 7b shows the PPL curves. The minimum test PPL reaches around 39, indicating acceptable response quality. The fluctuating training loss may be attributed to the nature of the dataset—specifically, the relatively small batch size. Training a generative model is inherently challenging, and variability in each batch can lead to fluctuations in the loss curve. However, our main objective is not to achieve state-of-the-art performance but to investigate the contributions of different multimodal interpersonal synchrony to empathetic response generation. Therefore, we maintained a batch size of 16 for consistency with the original CEM paper and applied it uniformly across all models in our study. In addition, although the training loss fluctuates, its overall downward trend suggests effective training. To prevent overfitting, we employed early stopping based on PPL.

**Figure 8:**
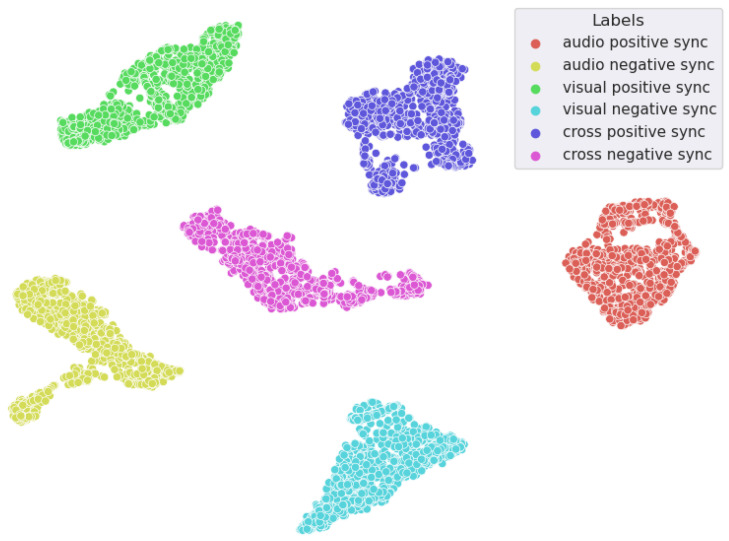
Visualization of positive and negative synchrony features in audio, visual, and cross-modalities using t-SNE.

In Figure 8, t-SNE has been applied to visualize different interpersonal synchrony features resulting from incorporating both positive and negative synchronies. The different features were clearly differentiated, indicating that qualitatively, the positive and negative synchrony features in the various modalities were successfully extracted.

Table 3 shows the performance of the models that used directional synchrony features. Comparing Table 2 and Table 3, we found that regardless of the modalities, when incorporating both positive and negative synchrony, the generated response is more similar to the ground truth response, and the generated response is more diverse, according to the BLEU, ROUGE, and Dist metrics.

- In the visual modality, there was an increase of 1.82 in BLEU, 0.0291 in ROUGE-1, 0.0156 in ROUGE-2, 0.0048 in Dist-1, and 0.0663 in Dist-2.
- In the audio modality, there was an increase of 1.81 in BLEU, 0.0305 in ROUGE-1, 0.0145 in ROUGE-2, 0.0046 in Dist-1, and 0.0459 in Dist-2.
- In the cross-modality, there was an increase of 0.79 in BLEU, 0.0092 in ROUGE-1, 0.0040 in ROUGE-2, 0.0055 in Dist-1, and 0.0441 in Dist-2.

The results demonstrate that positive synchrony and negative synchrony have been successfully integrated into the models and have benefited response generation. When comparing the results between {Pos sync vs. Neg sync vs. Pos & Neg sync}, we discovered that when using both positive and negative synchronies, the model performed better than when using only one synchrony, when considering the BLEU, ROUGE-1, ROUGE-2, Dist-1, and Dist-2 metrics. Consequently, both positive and negative synchronies are beneficial for the generation of responses.

Table 3 also indicates that considering positive or negative synchronies solely result in higher performance in the emotion classification task (i.e., EA metric) than both positive and negative synchronies in audio and cross-modalities.

- Audio modality shows that utilizing positive synchrony solely improved emotion classification accuracy by 1.34, while utilizing negative synchrony solely improved it by 0.72.
- Cross-modality shows that utilizing positive synchrony solely improved emotion classification accuracy by 0.44, while utilizing negative synchrony solely improved it by 0.09.

As we normally expect emotional classification performance to increase with new information, such results are very interesting. A possible reason may be that the integration of positive and negative interpersonal synchronies may lead the model to confusion. In contrast, solely considering the positive or negative interpersonal synchronies may contribute more to accurate detection of emotions in audio and cross-modalities. For instance, consider a communication scene. Both individuals plastered a fake smile. However, one speaker’s speech tone is extremely active, while his partner’s tone is extremely passive. When both people smile, positive synchrony is obtained, which indicates the responder should be happy. Negative synchrony obtained by using active tone and passive tone indicates the responder is sad. In such a situation, the model may be confused and classify emotions poorly. These results confirm the need to separate positive and negative synchronies.

We also performed the statistical effect size analysis for comparing the difference between the alternative models (with or without synchrony features). Specifically, we used Cohen’s d to measure the effect size based on BLEU, ROUGE-1, ROUGE-2, Dist-1, Dist-2, and EA metrics for the alternative models. To calculate Cohen’s d, we first normalized the evaluation values from both models (with and without synchrony features). Then, we subtracted the average value without synchrony features from the average value with synchrony features. Finally, this difference was divided by the standard deviation, as shown in Table 4. The effect size is large for each modality, indicating that the inclusion of synchrony features significantly contributes to the different performance of the model.

### 4.5. Synchronization Analysis Based on Empathetic Communication Mechanisms

Pretrained models are based on a publicly accessible dataset, Reddit, which contains over 8 million dialogues. Although such datasets are large, they are different from the datasets we used to generate the response. To migrate domain influence, we first labeled the ground truth using the pretrained model on the IEMOCAP dataset. Then, the pretrained model was fine-tuned with the IEMOCAP dataset. Based on Table 5, the fine-tuned model demonstrated high performance in the IEMOCAP domain; therefore, classifying the generated response with the fine-tuned model makes sense.

**Figure 9:**
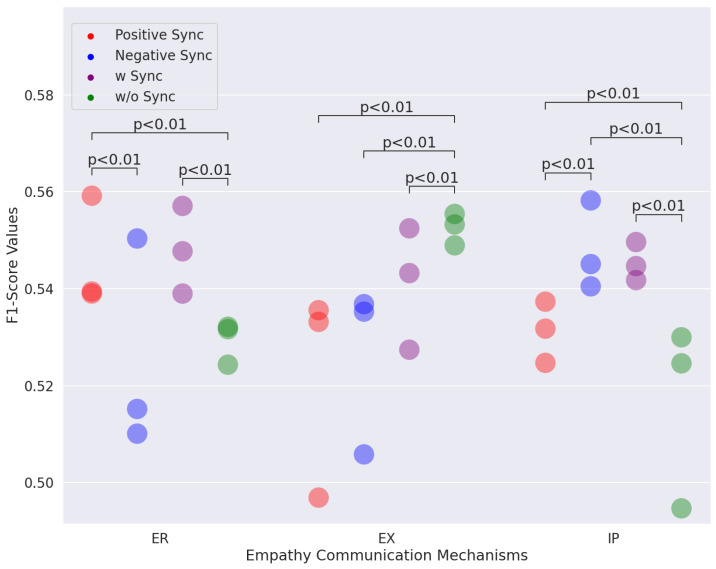
Empathy communication mechanisms’ performance comparison between positive synchronization, negative synchronization, and without synchronization.

Empathy identification results for the generated response incorporating multimodal directional synchrony are presented in Table 6. Additionally, as shown in Figure 9, we pooled F1-scores from all the modalities and compared them using the Wilcoxon signed-rank test when different synchronies were included. Since F1-scores are more suitable for evaluating performance than accuracy when data are imbalanced, we focused on F1-scores. We discovered several interesting results related to the classification accuracy of empathy communication mechanisms corresponding to the generated response.

Figure 9 shows that emotion reactions and interpretations distinguish positive (red points) from negative (blue points) synchronies. These results indicate that positive and negative synchronies are correlated with emotion reactions and interpretations in different ways.

Compared with conditions without synchrony, positive synchrony results in an average increase in the F1-scores of 1.65 for emotional reaction. As the previous finding indicated, positive synchrony is associated with the communication of emotions [68,69]. Such results may indicate that when two people mimic one another, they are more likely to express emotional reactions.

In comparison to conditions without synchrony, positive and negative synchronies significantly lower exploration F1-scores.

- Positive synchrony results in an average decrease in the F1-scores of 3.07 for exploration.
- Negative synchrony results in an average decrease in the F1-scores of 2.66 for exploration.

These results are in accordance with our intuition and make sense. Exploration represents the tendency of the responder’s response toward exploring unknown things from the Speaker in the cognitive aspect. Positive and negative synchronies are obtained when the two individuals’ behavior is similar or opposite. Take the opposite as an example: one individual actively speaks and moves forward to close the distance, while the other moves backward to escape. The responder is attempting to close the communication rather than asking a question.

Both positive and negative synchronicity result in significantly higher interpretation F1-scores.

- Positive synchrony results in an average increase in the F1-scores of 1.48 for interpretation.
- Negative synchrony results in an average increase in the F1-scores of 3.15 for interpretation.

Interpretation refers to the response within the cognitive process that incorporates information that is already known. Both positive and negative synchronies reflect the partner’s perception of the speaker’s information. No matter whether positive synchronicity or negative synchronicity is incorporated, the generated response reflects more related information that is known by communicators.

Based on these findings, we are able to shed light on how interpersonal synchronies relate to the emotional and cognitive aspects of communication with artificial technology.

## 5. Discussion

### Limitations

The theoretical model of empathy is complex. More comprehensive theoretical empathy models in psychology may not fully align with the empathy communication mechanisms we used, which evaluate empathy through exploration, emotional reactions, and interpretations. According to our knowledge, however, this is the current state-of-the-art computation model for empathy in computer science. Therefore, it is appropriate for initial exploration. We also acknowledge that the current empathy identification task has limitations. Even after the original three levels of classes for emotional reaction, exploration, and interpretation are merged into two levels, as explained in Section 4.1, the performance remains suboptimal.

Additionally, we acknowledge that the current results may not reflect sufficient rigor since we evaluated empathy identification using pseudo-labels. However, given the lack of multi-grained annotations in the original dataset, we believe that relying on pretrained tools is a justifiable approach. Furthermore, we recognize that the dataset used in this study may not be large enough for the generation task. This dataset, however, is the only public multimodal dataset available that is most similar to a natural two-person communication scenario. Our primary objective for this study is to compare models that incorporate different information, and this comparison can be performed even with a small dataset. Importantly, this is the first study to examine how different types of interpersonal synchrony contribute to the cognitive and emotional aspects of communication.

We acknowledge that the current model’s performance has room for improvement. Incorporating more advanced encoders, such as LLMs, could potentially enhance performance, and we plan to explore this in future work. However, LLMs are trained on large-scale datasets and may introduce external knowledge, which could affect the focus of our primary objective and potentially compromise the fairness of our comparisons. Additionally, to the best of our knowledge, few existing models for empathetic response generation incorporate both multimodal and interpersonal information. While a more advanced baseline model than CEM could further improve performance, we chose CEM for its integration of both affective and cognitive encoders. This dual-encoder structure makes it particularly suitable for analyzing empathy and aligns well with our research objectives, providing a robust framework for studying empathetic response generation.

## 6. Conclusions

This study aimed to explore how multimodal directional synchrony contributes to empathetic response generation. To achieve this, we developed a framework based on the CEM [21], integrating directional synchrony from audio, visual, and cross-modalities. We first validated that incorporating multimodal directional synchrony could improve empathetic response generation. Our results demonstrated that incorporating cross-modality synchrony, the most informative model, led to generated responses that are closer to the ground truth responses and more diverse. These findings confirm the effectiveness of directional interpersonal synchrony in the proposed model.

Based on this validation, we further explored how multimodal directional synchrony contributes to different components of the generated empathetic responses. Positive synchrony was associated with improved emotional reactions, reduced exploration, and enhanced interpretation. Negative synchrony was also linked to reduced exploration and enhanced interpretation.

Our findings underscore the importance of studying empathetic response generation in multimodal and interpersonal contexts. They also suggest potential correlations between the emotional and cognitive aspects of empathy and directional interpersonal synchrony. These insights help identify critical features, optimize models for better performance, and deepen our understanding of empathetic response patterns in communication scenarios involving artificial intelligence.

## Figures and Tables

**Table 1:** Statistical information for datasets.

	Train + Val	Test
Counts of total turns	3750	1118
Average utterance counts per turn	1.44	1.39
Average word counts per turn	21.74	22.13
Average duration (s)	6.65	6.36

**Table 2:** Performance comparison: text-only (MIME) vs. text-only (CEM) vs. multimodal approaches, across BLEU, ROUGE-1, ROUGE-2, Dist-1, Dist-2, and EA metrics.

Models	BLEU	ROUGE-1	ROUGE-2	Dist-1	Dist-2	EA
Text (MIME)	1.41	0.0930	0.0242	0.0120	0.0337	31.87
Text (CEM)	2.62	0.1236	0.0419	0.0232	0.0951	35.63
Text and Visual	3.17	0.1346	0.0470	0.0369	0.1842	38.76
Text and Audio	3.30	0.1369	0.0478	0.0363	0.1852	39.03
Text and Cross	4.71	0.1602	0.0649	0.0412	0.2314	41.72

**Table 3:** Performance evaluation: incorporating directional interpersonal synchronizations into multimodality.

Models		BLEU	ROUGE-1	ROUGE-2	Dist-1	Dist-2	EA
Text and Visual	Pos Sync	4.72	0.1628	0.0599	0.0378	0.2266	40.11
	Neg Sync	4.53	0.1609	0.0606	0.0410	0.2332	40.38
	Pos & Neg Sync	4.99	0.1637	0.0626	0.0417	0.2505	41.27
Text and Audio	Pos Sync	4.60	0.1624	0.0584	0.0378	0.2142	42.52
	Neg Sync	4.61	0.1554	0.0527	0.0363	0.1971	41.90
	Pos & Neg Sync	5.11	0.1674	0.0623	0.0409	0.2311	41.18
Text and Cross	Pos Sync	5.09	0.1599	0.0656	0.0459	0.2720	42.79
	Neg Sync	5.25	0.1668	0.0650	0.0419	0.2448	42.44
	Pos & Neg Sync	5.50	0.1694	0.0689	0.0467	0.2755	42.35

**Table 4:** Statistical effect size of the difference between the alternative models (with or without synchrony features).

Model	Cohen’s d
Text and Visual	1.89
Text and Audio	1.86
Text and Cross	1.68

**Table 5:** Fine-tuning results for the empathy communication identification task of IEMOCAP.

Models	Emotional Reactions		Explorations		Interpretations	
	**Acc**	**F1**	**Acc**	**F1**	**Acc**	**F1**
Finetune Results	97.05	96.32	99.29	99.06	97.68	96.89

**Table 6:** Empathy identification evaluation: incorporating directional interpersonal synchronization into multimodality.

Models		Emotional Reactions		Explorations		Interpretations	
		**Acc**	**F1**	**Acc**	**F1**	**Acc**	**F1**
Text and Visual	Pos Sync	64.28	53.90	71.44	53.32	80.84	52.47
	Neg Sync	64.01	51.52	70.55	53.68	81.92	54.51
	With Pos & Neg Sync	65.00	53.90	70.37	54.32	81.29	54.96
	Without Sync	66.25	52.43	71.35	55.33	77.98	49.47
Text and Audio	Pos Sync	63.35	53.94	71.71	49.69	78.42	53.73
	Neg Sync	61.50	51.01	72.52	53.53	80.21	55.82
	With Pos & Neg Sync	64.91	55.71	71.62	52.74	80.21	54.18
	Without Sync	71.80	53.22	66.52	54.90	79.41	52.46
Text and Cross	Pos Sync	68.22	55.92	69.92	53.56	80.04	53.18
	Neg Sync	69.02	55.04	68.31	50.58	80.04	54.05
	With Pos & Neg Sync	68.13	54.77	70.64	55.25	81.02	54.47
	Without Sync	65.53	53.17	72.07	55.54	78.51	53.00

## Data Availability

Link to IEMOCAP dataset: https://sail.usc.edu/iemocap/ (accessed on 4 December 2024).
